# Supplementary material for: Energy Storage Application of CaO/Graphite Nanocomposite Powder Obtained from Waste Eggshells and Used Lithium-Ion Batteries as a Sustainable Development Approach
Source: Nanomaterials (Basel). 2024 Jun 30;14(13):1129. doi: 10.3390/nano14131129 (PMC11243355; doi:10.3390/nano14131129)
Supplement: Supplementary file 1 [file nanomaterials-14-01129-s001.zip › nanomaterials-3079115-supplementary.pdf]

Energy Storage Application of CaO/Graphite Nanocomposite Powder obtained from Waste Eggshells and Used Lithium-ion Batteries as a Sustainable Development Approach

Kathalingam Adaikalam[1], Aviraj M. Teli[2], Karuppasamy Pandian Marimuthu[3], Sivalingam Ramesh[4], Hyungyil Lee[3], Heung Soo Kim[4], Hyun-Seok Kim[2*]

[1]*Millimeter-wave Innovation Technology (MINT) Research Center, Dongguk University-Seoul, Seou-04620, Republic of Korea.*

[2]*Division of Electronics and Electrical Engineering, Dongguk University-Seoul, Seoul 04620, Republic of Korea*

[3]*Department of Mechanical Engineering, Sogang University, Seoul 04107, Republic of Korea*

[4]*Department of Mechanical, Robotics and Energy Engineering, Dongguk University-Seoul, Seoul-04620, Republic of Korea*

* Corresponding author, E-mail: hyunseokk@dongguk.edu

Abstract

The reuse of waste materials has recently become appealing due to the pollution and cost reduction factors. Using waste materials can reduce environmental pollution and product costs, thus promoting sustainability. Approximately 95% of calcium carbonate-containing waste eggshells end up in landfills, unused. These eggshells, a form of bio-waste, can be repurposed as catalytic electrode material for various applications, including supercapacitors, after being converted into CaO. Similarly, used waste battery electrode materials pose environmental hazards if not properly recycled. Various types of batteries, particularly lithium-ion batteries, are extensively used worldwide. The recycling of used lithium-ion batteries has become less important considering its low economic benefits. This necessitates finding alternative methods to recover and reuse the graphite rods of spent batteries. Therefore, this study reports the conversion of waste eggshell into calcium oxide by high-temperature calcination and extraction of nanographite from spent batteries for application in energy storage fields. Both CaO and CaO/graphite were characterized for their structural, morphological, and chemical compositions using XRD, SEM, TEM, and XPS techniques. The prepared CaO/graphite nanocomposite material was evaluated for its efficiency in electrochemical supercapacitor applications. CaO and its composite with graphite powder obtained from used lithium-ion batteries demonstrated improved performance compared to CaO alone for energy storage applications. Using these waste materials for electrochemical energy storage and

conversion devices results in cheaper, greener, and sustainable processes. This approach not only aids in energy storage but also promotes sustainability through waste management by reducing landfills.

Keywords: Chicken eggshell**;** CaO**;** Graphite rods; Waste Utilization; Supercapacitor; Energy storage; Sustainability

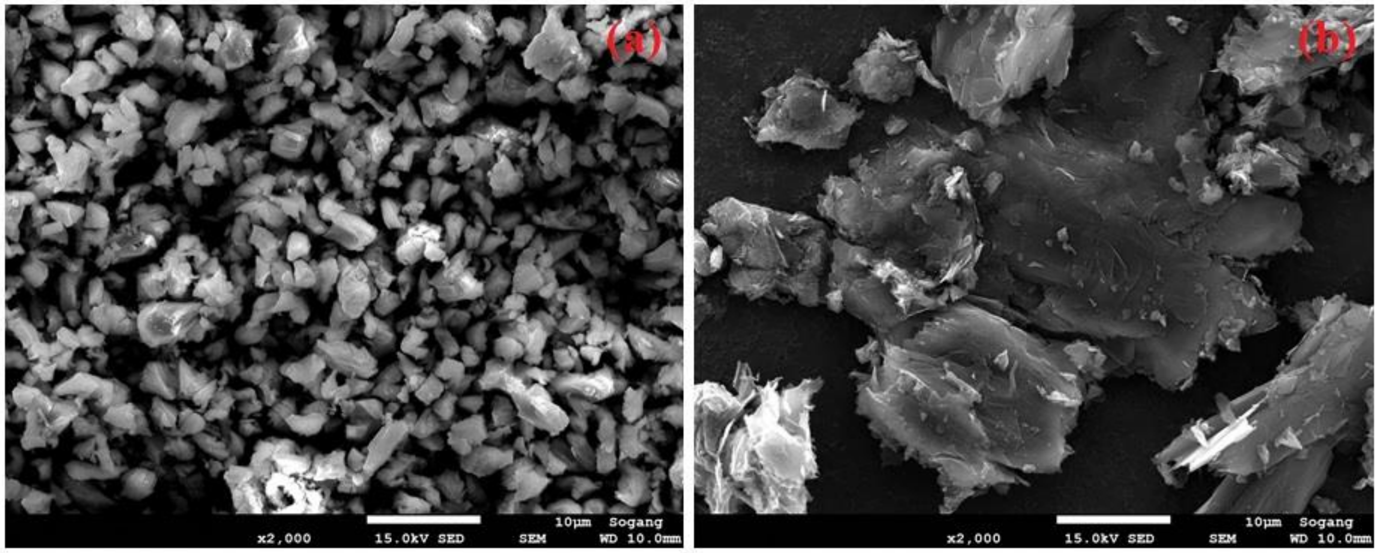

Figure S1. SEM images of (a) CaO NPs and (b) graphite powder

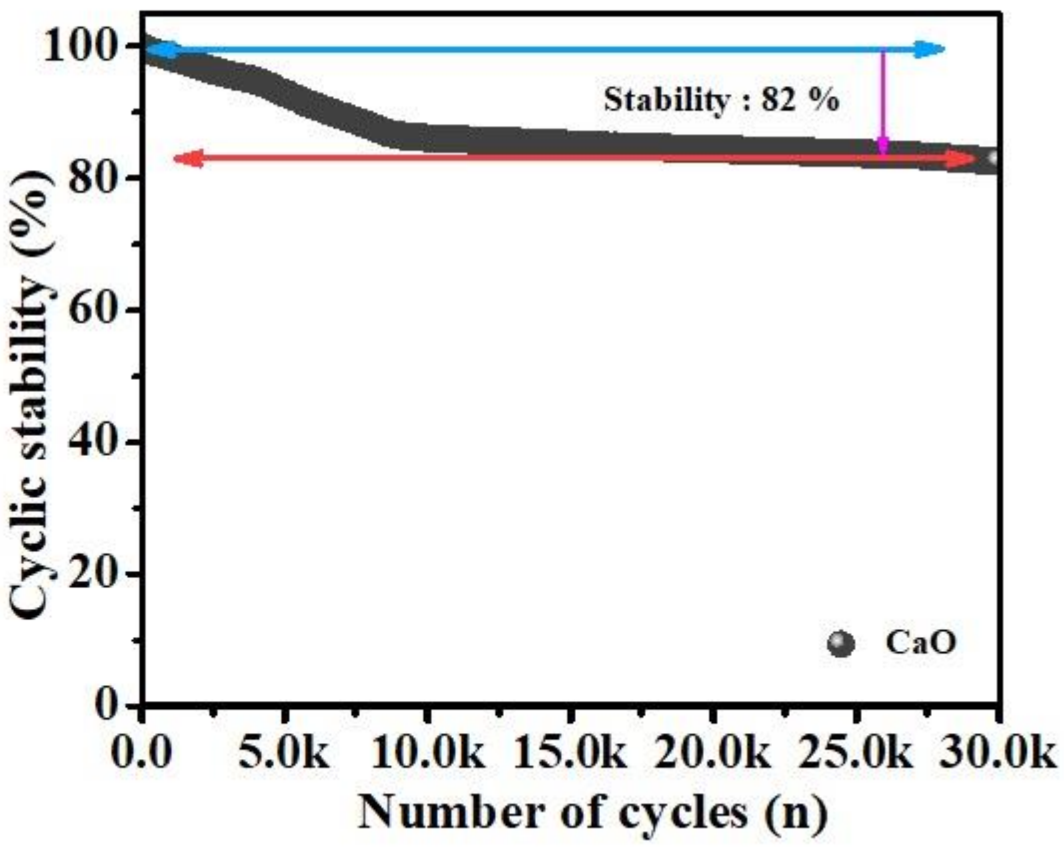

Figure S2. Cyclic stability of CaO nanoparticles
